# Galectin‐1 as a therapeutic strategy and a biomarker for Alzheimer's disease

**DOI:** 10.1002/alz70855_096005

**Published:** 2025-12-23

**Authors:** Carlos Javier Pomilio, Ángeles Vinuesa, Jessica L Presa, Amanda Cano, Juan Beauquis, Gabriel A Rabinovich, Flavia Saravia

**Affiliations:** ^1^ CONICET, Buenos Aires, Argentina; ^2^ University of Buenos Aires, Buenos Aires, Argentina; ^3^ CONICET, Buenos Aires, Buenos Aires, Argentina; ^4^ Research Center and Memory Clinic, Fundació ACE Institut Català de Neurociències Aplicades ‐ Universitat Internacional de Catalunya (UIC), Barcelona, Spain; ^5^ Ace Alzheimer Center Barcelona, Barcelona, Spain; ^6^ CIBERNED, Network Center for Biomedical Research in Neurodegenerative Diseases, National Institute of Health Carlos III, Madrid, Spain; ^7^ CONICET, Ciudad Autónoma de Buenos Aires, Buenos Aires, Argentina; ^8^ University of Buenos Aires, Ciudad Autónoma de Buenos Aires, Buenos Aires, Argentina

## Abstract

**Background:**

The rising prevalence of Alzheimer's disease (AD), the most preponderant form of dementia, represents a global health emergency. Early detection and novel therapeutic strategies are urgently needed due to the gradual progression of this neurodegenerative condition. Here, we identify for the first‐time elevated levels of Galectin‐1 (Gal‐1), a conserved immunomodulatory lectin, in the cerebrospinal fluid of AD patients, correlating with classic AD biomarkers since prodromal stages. Notably, raised Gal‐1 levels in cerebrospinal fluid were a significant risk factor for the progression from mild cognitive impairment to AD dementia.

**Methods:**

The strong labeling of Gal1+ in astrocytes from post‐mortem AD brain tissue and in the hippocampus of transgenic PDAPPJ20 mice prompted us to investigate its potential role in the pathology. In vitro and in vivo studies revealed that Gal‐1 specially modulated microglia, a key player in AD pathogenesis, by inducing a deactivated state and restoring Aβ phagocytosis.

**Results:**

The administration of supraphysiological levels of Gal‐1 to aged AD mice resulted in a notable reduction in amyloid burden and a robust cognitive improvement. The transcriptomic analysis following Gal‐1 therapy revealed recovered neuronal signaling pathways and an immune profile resembling that of age‐matched controls in the hippocampus, which aligns with the observed behavioral recovery. These results suggest that Gal‐1 facilitates Aβ clearance while fostering a neuroprotective and less inflammatory environment in the brain.

**Conclusion:**

Our novel findings, which include brain tissue and data from a large cohort of patients, in vitro and in vivo approaches, have identified a glycosylation‐dependent pathway as a promising avenue for therapeutic intervention in AD.

